# Predicting the outcome for COVID-19 patients by applying time series classification to electronic health records

**DOI:** 10.1186/s12911-022-01931-5

**Published:** 2022-07-17

**Authors:** Davi Silva Rodrigues, Ana Catharina S. Nastri, Marcello M. Magri, Maura Salaroli de Oliveira, Ester C. Sabino, Pedro H. M. F. Figueiredo, Anna S. Levin, Maristela P. Freire, Leila S. Harima, Fátima L. S. Nunes, João Eduardo Ferreira, HCFMUSP COVID-19 Study Group, Geraldo Busatto, Eloisa Bonfá, Edivaldo Utiyama, Aluisio Segurado, Beatriz Perondi, Anna M. Morais, Amanda Montal, Solange Fusco, Marjorie Fregonesi, Marcelo Rocha, Izabel Marcilio, Izabel C. Rios, Fabiane Y. O. Kawano, M. Amelia de Jesus, Esper G. Kallas, Carolina Marmo, Clarice Tanaka, Heraldo P. de Souza, Julio F. M. Marchini, Carlos Carvalho, Juliana C. Ferreira, Thais Guimaraes, Carolina S. Lazari, Alberto J. S. Duarte, M. Cristina P. B. Francisco, Silvia F. Costa

**Affiliations:** 1 Laboratory of Computer Applications for Health Care, School of Arts, Sciences and Humanities, Universidade de São Paulo, São Paulo, Brazil; 2 Division of Infectious Diseases, Faculdade de Medicina, Universidade de São Paulo, São Paulo, Brazil; 3 Department of Infection Control, Hospital das Clínicas, Universidade de São Paulo, São Paulo, Brazil; 4 Núcleo de Vigilância Epidemiológica, Hospital das Clínicas, Faculdade de Medicina, Universidade de São Paulo, São Paulo, Brazil; 5 Clinical Director’s Office, Hospital das Clínicas, Faculdade de Medicina, Universidade de São Paulo, São Paulo, Brazil; 6 Computer Science Department, Institute of Mathematics and Statistics, Universidade de São Paulo, São Paulo, Brazil; 7 Hospital das Clínicas da Faculdade de Medicina, Universidade de São Paulo, São Paulo, Brazil

**Keywords:** COVID-19, Outcome prediction, Vital signs, Time series classification

## Abstract

**Background:**

COVID-19 caused more than 622 thousand deaths in Brazil. The infection can be asymptomatic and cause mild symptoms, but it also can evolve into a severe disease and lead to death. It is difficult to predict which patients will develop severe disease. There are, in the literature, machine learning models capable of assisting diagnose and predicting outcomes for several diseases, but usually these models require laboratory tests and/or imaging.

**Methods:**

We conducted a observational cohort study that evaluated vital signs and measurements from patients who were admitted to Hospital das Clínicas (São Paulo, Brazil) between March 2020 and October 2021 due to COVID-19. The data was then represented as univariate and multivariate time series, that were used to train and test machine learning models capable of predicting a patient’s outcome.

**Results:**

Time series-based machine learning models are capable of predicting a COVID-19 patient’s outcome with up to 96% general accuracy and 81% accuracy considering only the first hospitalization day. The models can reach up to 99% sensitivity (discharge prediction) and up to 91% specificity (death prediction).

**Conclusions:**

Results indicate that time series-based machine learning models combined with easily obtainable data can predict COVID-19 outcomes and support clinical decisions. With further research, these models can potentially help doctors diagnose other diseases.

## Background

Coronavirus disease 2019 (COVID-19) has affected approximately 350 million people globally, having caused more than 5 million deaths as of January 25, 2022. In Brazil, so far, there have been more than 24 million diagnosed cases and 622,000 deaths [1]. The disease has different forms of presentation, from mild to very severe, with variable death rates worldwide (0.1 to 19.3%).

Unfavorable outcome of COVID-19 has various determinants, including populational and individual factors [2], and the virus itself. During the pandemic, we observed the emergence of different variants of SARS-CoV-2, which were responsible for the waves of infection.

In Brazil, there was a first increase in the number of COVID-19 cases between April and May 2020, related to the introduction and initial spread of the virus. A second wave started in December, 2020, reaching its peak in March, 2021 due to the emergence of the novel SARS-CoV-2 gamma variant (P.1) [3], when more than 90,000 cases per day were recorded. Due to this significant occurrence, we decided to perform the data analysis in two stages, roughly corresponding to the two waves. Determining risk factors or individual characteristics that help predict the outcome of patients may be one of the strategies to deal with COVID-19.

Hospital das Clínicas (HC) is a public teaching hospital located in São Paulo, Brazil. It comprises seven buildings with 2200 beds and approximately 22,000 employees. The hospital was designated by the São Paulo State government to receive the severe cases of COVID-19. The Central Institute (CI) is an 11-floor building with 6000 healthcare workers designated from March 2020 through August 2020 to receive all the COVID-19 cases referred to the hospital. It included an emergency unit, 300 ICU beds, 300 beds in regular wards, and was entirely dedicated to COVID-19 care [4]. After this period, the hospital continued to receive cases spread out in all of its seven institutes, in COVID-19-designated areas. This scenario shows itself rich enough to acquire a large volume of data from patients with different characteristics, divided into two periods of time, which were the first and the second waves mentioned above.

Machine learning has been used in health care applications to both diagnose diseases and predict patients’ outcomes. Deep learning neural networks have been used to aid diagnosis based on images of diseases such as breast cancer [5], skin cancer [6], and histopathologic cancer [7]. Other data such as vital signs, patient history, and laboratory tests have also been used as input for machine learning models. Recurrent neural networks, for example, were used to diagnose acute kidney injury based on vital signs, prescriptions, laboratory tests, admission dates, and other data available in electronic health records (EHRs) [8]. The prediction of the outcome of a patient has also been assisted by machine learning. For septic patients, models such as feed forward neural networks (based on patient’s history of diseases) [9] and recurrent neural networks (based on vital signs and heart rate variability) [10, 11] were used to predict their outcome. For COVID-19 a Time Aware Long Short-Term Memory neural network (T-LSTM) was recently used to predict the patients’ outcome based on bio markers present in blood samples [12].

Although vital signs are already used to predict outcomes related to COVID-19, the predictions presented in the literature take into consideration laboratory tests and images in addition to these signs. Since vital signs are routinely used in healthcare and do not require expensive equipment for their acquisition, vital signs-based models can be more broadly adopted. Additionally, time series-based models were under explored in the literature within this context. In this paper, we use univariate and multivariate time series to represent COVID-19 patients’ vital signs and other simple routine measurements to build classifiers capable of predicting their outcomes. Most of these data can be routinely collected for any patient and are easily available in any level of healthcare.

The main contributions of this study are: providing machine learning models capable of predicting COVID-19 patients’ outcomes with up to 81% accuracy in the first day of hospitalization; using easily obtainable vital signs and measurements to predict outcomes, without the need of laboratory tests or imaging; and the possibility of identifying which vital signs or measurements directed the models to a certain prediction, helping doctors make clinical decisions.

Besides this background, in this paper “Materials and methods” section details the datasets, data models, and time series models we proposed; “Results” section presents the results of multiple time series models tests; “Discussion” section discusses these results, highlighting advantages and limitations of the models; “Conclusions” section concludes this paper, by presenting the main contributions and future research possibilities.

## Materials and methods

This observational cohort study evaluated patients who were admitted to Hospital das Clinicas between March 2020 and October 2021 due to COVID-19. The criteria considered to identify patients are described in “Datasets” section.

**Fig. 1:**
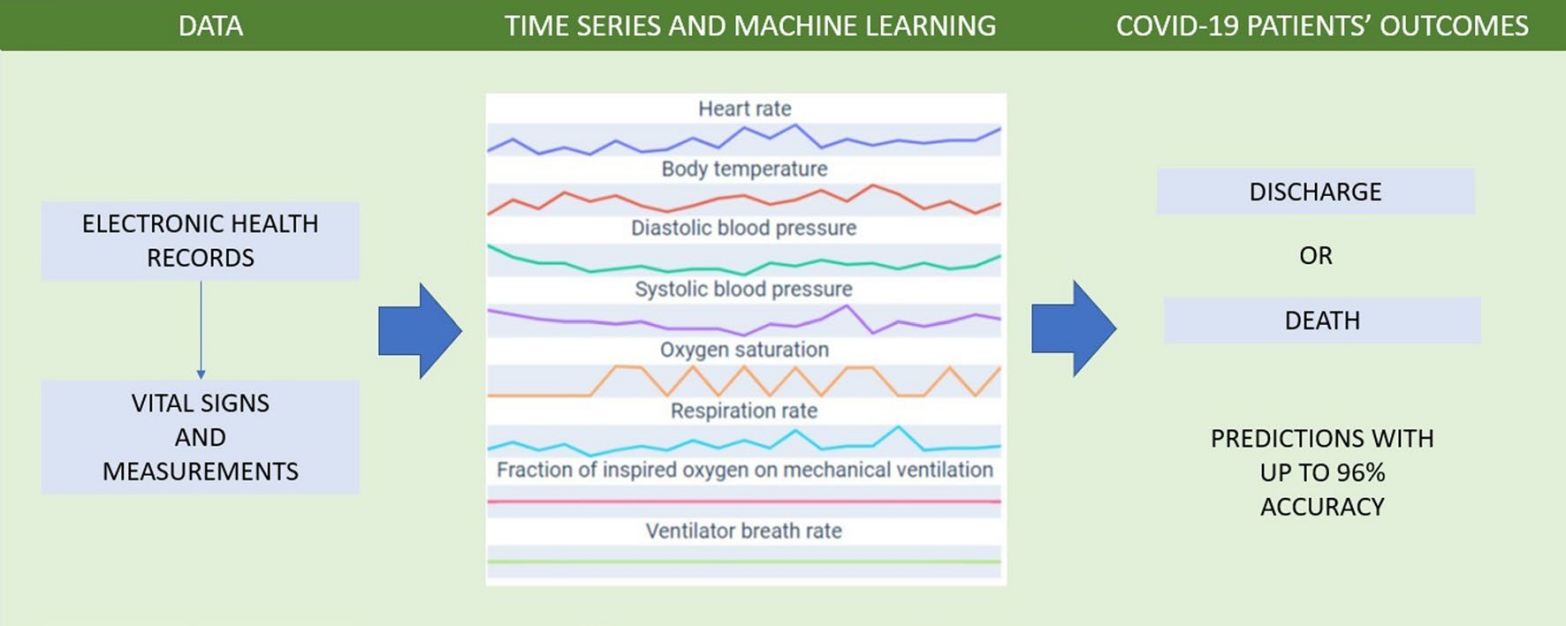
Methods for predicting severe COVID-19 patients’ outcome

A selection of vital signs and routine measurements were extracted from COVID-19 patients’ EHRs. The data were used to create multivariate and univariate time series representing each patient’s evolution during hospitalization. These time series were used to train and test classifiers based on time series transformations and random convolutional kernels, as described in “Training the models” section. The trained models were able to predict a patient’s outcome (death or discharge) when at least one day of electronic medical records is available (Fig. 1).

“Datasets” section describes the datasets used for training and testing time series models. In “Preprocessing the data” section we detail the data preprocessing step. “Modelling the data” section explains the different data models that were applied to the datasets. “Training the models” and “Predicting the outcome for COVID-19 patients” sections present, respectively, the training and the testing of the time series classifiers.

### Datasets

The inclusion criteria to compose the HC’s datasets with EHRs from patients with COVID-19 were:

- The patient should present at least one of the following symptoms: cough, fever, shortness of breath, sudden onset of anosmia, ageusia or dysgeusia;
- The patient should fulfill at least one of the following evidence:
  - radiological evidence showing lesions compatible with COVID-19 (e.g. bilateral, peripheral ground-glass opacities);
  - positive RT-PCR test or antigen test for SARS-CoV-2 in a clinical specimen (oro-nasopharyngeal swab or bronchoalveolar lavage).

Table 1 shows eight different vital signs and measurements extracted from 6692 COVID-19 patients’ electronic medical records (EHRs): heart rate, respiration rate, systolic blood pressure, diastolic blood pressure, oxygen saturation, body temperature, and, when applicable, fraction of inspired oxygen on mechanical ventilation and ventilator breath rate. Since each vital sign and measurement was associated with a date, it is possible to observe a patient’s evolution during hospitalization.

**Table 1:** Vital signs and measurements available in EHRs

Vital sign/measurement	Number of patients (%)
Heart rate	6691 (99.98)
Body temperature	6689 (99.95)
Diastolic blood pressure	6688 (99.94)
Systolic blood pressure	6688 (99.94)
Oxygen saturation	6686 (99.91)
Respiration rate	6644 (99.28)
Fraction of inspired oxygen on mechanical ventilation	3161 (47.23)
Ventilator breath rate	2746 (41.03)

The second column refers to the number of patients who had available data for each vital sign/measurement

Besides the vital signs/measurements, additional data were extracted from EHRs: patients’ final outcome (death or discharge), age, gender, and date of hospital discharge. In our approach, the final outcome is the situation of a patient at the end of the hospitalization period.

The EHRs were divided into two datasets. The first one contains 3394 patients’ EHRs collected during the first wave of COVID-19, from March 2020 to December 2020. The second one contains 2238 patients’ EHRs collected during the second wave of COVID-19, from January 2021 to October 2021.

The demographic data concerning the patients present in the databases are shown in Table 2. The mortality rate decreased from 33.29% in the first wave to 26.99% in the second wave. The opposite trend can be observed with the mean age of the patients: increased from 51 to 57 years-old. The mean hospital stay length had a slight decrease from 20 days in the first wave to 17 days in the second wave.

**Table 2:** Demographic data of the patients admitted to Hospital das Clínicas in each COVID-19 wave, from March 2020 to October 2021

	First wave	Second wave
Number of patients	3394	2238
Female patients	1508 (44.43%)	1041 (46.51%)
Age—mean (SD)	51 (28) years	57 (23) years
Days of hospitalization—mean (SD)	20 (21.09) days	17 (15.58) days
Patients on ICU	2207 (65.03%)	1694 (75.69%)
Number of deaths	1130 (33.29%)	604 (26.99%)
Period analyzed	03/2020 to 12/2020	01/2021 to 10/2021

*SD* standard deviation, *ICU* intensive care unit

### Preprocessing the data

The dataset was formed by combining vital signs/measurements data and additional information about the patients (i.e., patient’s outcome, discharge date, age, gender, etc.). Each sample in the dataset is a multivariate time series, which are represented by eight lists of vital signs/measurements recorded in a single day. Each list can have a variable number of values. For example, a patient admitted for three and a half days generates four multivariate time series, since there are four different dates in her EHRs. Each time series has eight lists, one for each vital sign/measurement. The length of each list is determined by the number of samples available, i.e., how many times a vital sign/measurement was taken. If a patient has ten occurrences recorded for each vital sign/measurement in a single day, then the multivariate time series of the day will have 80 values, i.e, the number of occurrences (ten) multiplied by the eight vital signs/measurements.

Due to the method we used for time series classification (“Training the models” section), there was no need to normalize data. However, this method requires that every multivariate time series in the dataset have the same length. This requirement was satisfied by making the length of every list of vital signs/measurements be the same as the biggest list in the dataset. Consequently, every day of hospitalization was equalized in terms of length, i.e., each list was filled to reach the maximum number of vital signs/measurements taken for a patient of the dataset. For example, if the patient with the most vital signs/measurements has 40 heart rate readings registered and a lesser amount of readings for other vital signs, then every patient must have 40 values for every vital sign/measurement in all hospitalization days. The vital signs/measurements with less than 40 readings will be filled with the average of the occurrences until they reach 40 values. Consequently, all multivariate time series will have the same length.

Two approaches were tested in this step. First, each list fill was performed by using the average of the vital sign/measurement available. This value was repeated to complete each list until they reached the same length. Second, we tested to fill each list with zeros. Since no significant difference was detected in the accuracy of the predictions, the average was used to avoid confusion between zeros, missing data, and, consequently, any relation with a patient’s death.

### Modelling the data

**Fig. 2:**
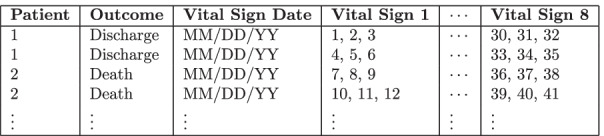
Example of independent days of hospitalization data modelling

**Fig. 3:**
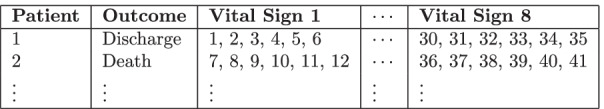
Example of complete hospitalization history data modelling

Two different data models were considered in this work:

1. independent days of hospitalization (Fig. 2): each time series represents the recorded vital signs/measurements of a patient in a single day; this data model does not discriminate between patients, i.e., different days of the same patient or of distinct patients are independent samples.
2. complete hospitalization history (Fig. 3): each time series represents the recorded vital signs/measurements of a patient during the entire hospitalization, i.e, each sample is related to a patient’s history.

Each data model was used with both univariate and multivariate time series. An univariate time series contains all the values of only a single vital sign/measurement (e.g., heart rate or ventilation breath rate) recorded in a single day or during the patient’s entire hospitalization. A multivariate time series contains the values of the eight vital signs/measurements recorded in a single day or during the entire patient’s hospitalization.

The preprocessing steps described in “Preprocessing the data” section were sufficient for the first data model, but did not address the particularities of the second data model: although every day of hospitalization had the same number of vital signs/measurements across the dataset, the hospital stay varied from 1 to 262 days. Consequently, the length of each time series varied according to the days of hospitalization. This difference was eliminated by completing the “missing” days with zeros. Initially, all the time series were filled with zeros until they represented 262 hospitalization days for each patient. However, the length of the time series increased the execution times to the point of impracticality. To avoid this problem, only the first 120 days of hospitalization were used in the models.

As an example, considering both data model categories (univariate and multivariate), a patient hospitalized for 3 days would have:

- considering independent days of hospitalization:
  - three multivariate time series: each one formed by the same number of readings for all the eight vital signs.
  - 24 univariate time series: eight univariate time series per day, each time series formed by the readings of a single vital sign.
- considering the complete hospitalization history:
  - one multivariate time series formed by all the vital signs readings recorded during the hospitalization.
  - eight univariate time series: one time series per vital sign, each one formed by all the readings of a single vital sign recorded during the entire hospitalization.

The implementation of the models is detailed in “Training the models” section. The performance of the models with different amounts of data (days of hospitalization) are presented in “Results” section.

### Training the models

The preprocessed data was used as input for MiniRocket [13] algorithm, a method for time series classification. This method uses random convolutional kernels to transform time series and uses the transformed time series as input for a linear classifier that does the actual prediction.

There are several state-of-the-art methods for time series classification, such as LSTM-FCN, cBOSS, Proximity Forest, Canonical Interval Forest (CIF), Temporal Dictionary Ensemble (TDE), InceptionTime, Rocket, TS-CHIEF, HIVE-COTE/TDE, etc. MiniRocket authors conducted benchmarks [13, 14] with the popular UCR Time Series Classification Archive [15] datasets, including long time series and datasets with a high number of instances. The authors observed better accuracy than most of the state-of-the-art methods above mentioned. The only exceptions were TS-CHIEF and HIVE-COTE/TDE, which achieved slightly higher accuracies. However, MiniRocket used only a little fraction of the execution time when compared to other methods. For example, the computing time spent with the training and testing with 109 UCR datasets is more than two weeks for TS-CHIEF and eight minutes for MiniRocket [13]. Additionally, MiniRocket is compatible with both univariate and multivariate time series, there are no parameters to adjust, data normalization is not required and the results are almost deterministic. The reasons presented justify the choice for this method.

With MiniRocket, time series are transformed by a fixed set of 84 convolutional kernels. This transformation is made with additions (instead of multiplications) to reduce execution time. The transformations produce 10,000 features for each original time series. These features are called PPV (proportion of positive values). Using the PPVs obtained for each time series, a ridge regression classifier is used to predict a class [13]. In this study we consider two classes: a positive class representing a patient’s discharge and a negative class representing a patient’s death. Thus, we have a binary classification where the method output must predict, from the vital signs/measurements, the patient’s final status.

As the observed mortality rates were 33.29% and 26.99% during the first and second waves, respectively, the datasets have unbalanced class (outcome) distribution. We used a stratified cross-validation strategy to ensure that all data are used both to train and to test the machine learning models, but avoiding overfitting and possible distortions in the accuracy due to different class distributions in each fold.

**Fig. 4:**
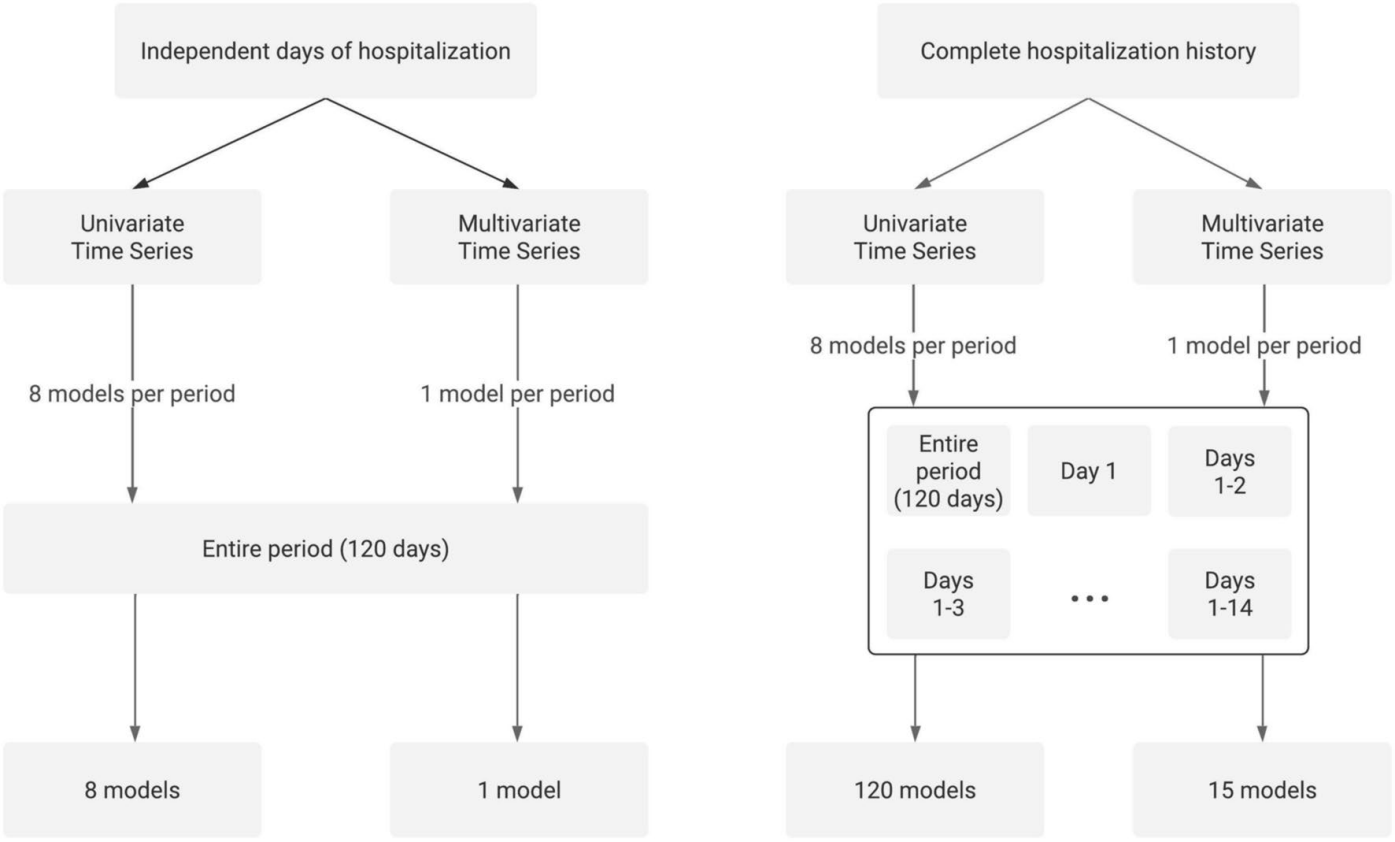
Overview of the 144 time series models that were trained and tested in this work

We trained and tested 144 models, combining different data models and time series, as shown in Fig. 4. For independent days of hospitalization, eight univariate models were trained and tested (one for each vital sign/measurement) and one multivariate model was trained and tested. For the complete hospitalization history, one multivariate model was trained and tested with all available data and 14 multivariate models were trained with partial data. As for univariate models, eight models were trained and tested with all available data and 120 were trained and tested with partial data. The high number of models were necessary to conduct the experiments.

For the second data model, which considers the complete patient’s hospitalization history, two experiments were conducted:

- Experiment 1: train with complete hospitalization history and test with partial hospitalization history;
- Experiment 2: train and test with partial hospitalization history, from the first day until the 14th day of hospitalization;

The first experiment consisted in using part of the dataset for training and the other part for testing. The testing considered data until the 14th day of hospitalization. This test was also conducted with independent days of hospitalization.

In the second experiment, both training and testing were done with partial data: train and test with data until the ith hospitalization day, from the first day until the 14th (i.e., train with complete data and test with data of the first day, then test with data of the first two days, then test with data of the first 3 days, and so on).

Experiment 1 was designed to indicate whether using the complete hospitalization history as a multivariate time series would result in better predictions and to simulate a real world use case (i.e., predict the outcome for a patient currently hospitalized). Experiment 2 was designed to indicate whether the inclusion of more data would result in better predictions.

By using the same datasets with multiple data models it is possible to compare the performance of the time series models with all the available data versus the performance of these models with limited data. It is also possible to measure the impact of applying univariate and multivariate approaches to the same problem and datasets.

### Predicting the outcome for COVID-19 patients

We used the scikit-learn [16] implementation of the stratified k-fold cross-validation strategy to train and test all models with threefolds. This method divides the dataset in *k* folds taking into consideration the proportion of the classes. In our case, the datasets are unbalanced with around 70% of discharge outcomes. Thus, this method produces folds that maintain the proportion between the classes, i.e., each fold is composed by 70% of instances related to discharges and 30% of instances related to deaths. Each fold is then used to both train and test each model. This cross-validation strategy minimizes the possibility of overfitting our models by using all available data in both training and testing steps, without the risk of using folds of the dataset that contains just one of the classes.

**Fig. 5:**
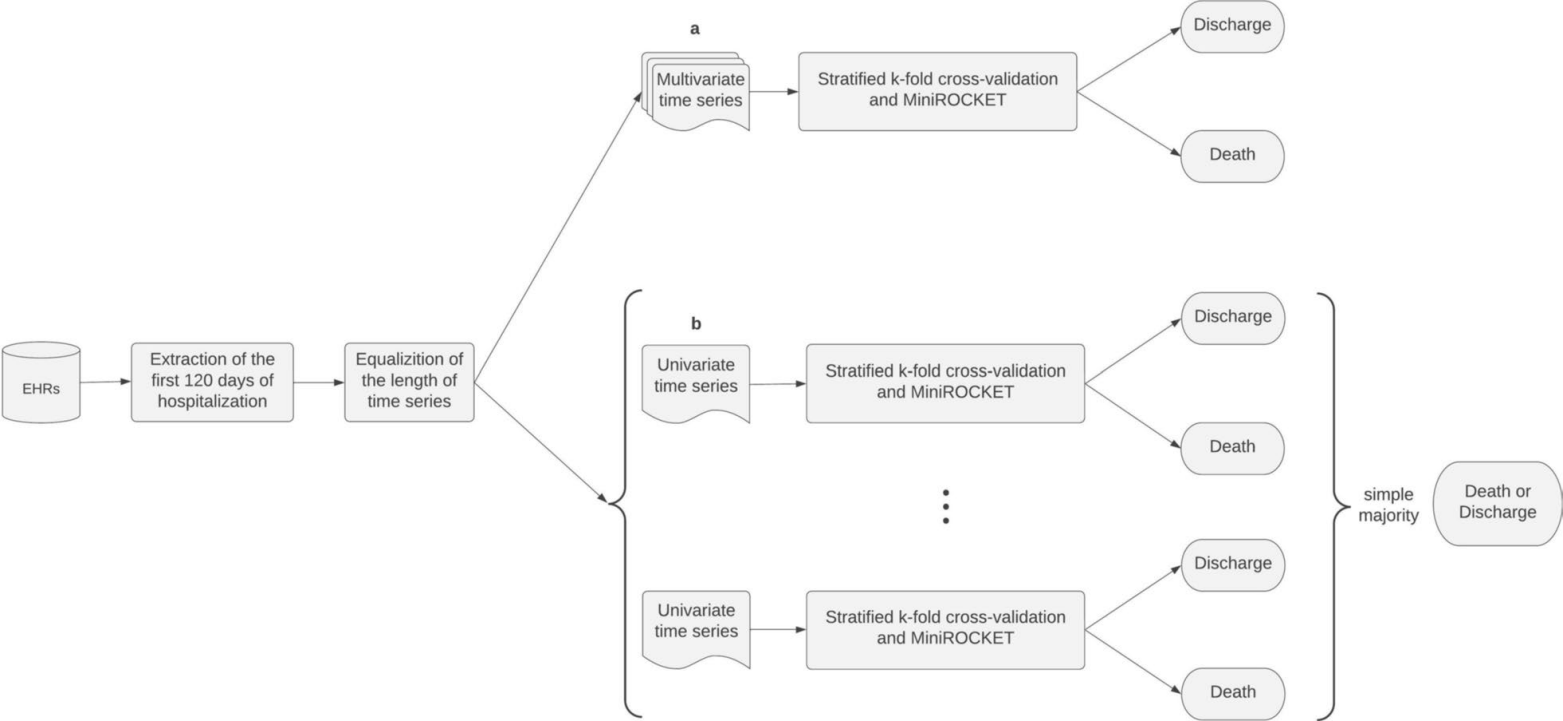
Flowchart of the univariate and multivariate time series classification method

The multivariate time series models use the preprocessed EHRs as input (see “Preprocessing the data” section), transform the time series, execute convolutions, and predict the outcome for each patient (Fig. 5a).

The univariate time series models function similarly to the multivariate ones but, by definition, can only consider a single vital sign/measurement. To predict the patient’s outcome, we created ensemble classifiers that combine eight univariate models (one for each vital sign/measurement) and use simple voting to predict the patient’s outcome (Fig. 5b). Each univariate time series predicts an outcome for a patient and the most predicted outcome (simple majority) is considered the outcome of the ensemble. A weighted average was tested, but no significant difference in the ensemble accuracy was observed.

For each model tested, six metrics were collected: accuracy, sensitivity, precision, F1 score, specificity, and negative predictive value. Since this is a binary time series classification problem, the discharge outcome was considered the positive class and it was associated with sensitivity, precision, and the F1 score. The death outcome was considered the negative class and it was associated with specificity and negative predictive value.

## Results

This section presents the results of multiple combinations of data models and time series models in predicting the outcome of COVID-19 patients. Results for univariate time series models are shown in “Univariate time series models” section, multivariate time series models in “Multivariate time series models” section and a comparison between the models is presented in “Comparisons between models” section.

### Univariate time series models

An ensemble of eight univariate time series models was trained and tested with the data models for both COVID-19 waves. Each model was trained with data recorded for a single vital sign/measurement, as detailed in “Modelling the data” section. For each patient, the outcome predicted by the ensemble is the one predicted by the majority of the univariate models.

#### Independent days of hospitalization

Table 3 shows the average metrics collected during the tests. When tested with independent days of hospitalization, the ensemble achieved a higher accuracy with the second wave dataset. In both datasets, the ensemble was capable of correctly predicting most patients’ discharge, as the high sensitivity and precision indicate. However, specificity was low for the second wave, showing that the ensemble had difficulty predicting patients’ deaths in this dataset.

##### Experiment 1: tests by days of hospitalization with univariate time series models

**Fig. 6:**
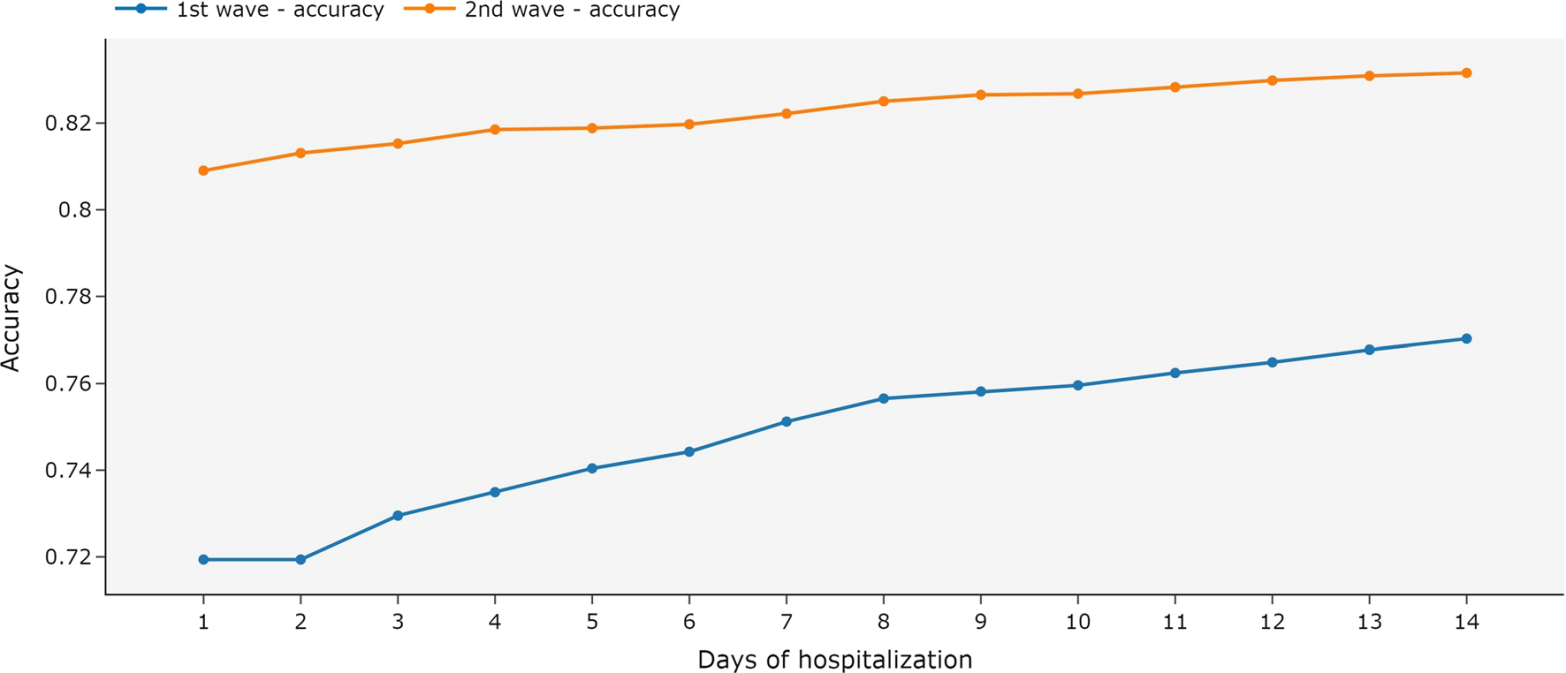
Metrics for an ensemble of MiniRocket models using independent days of hospitalization and univariate time series. The ensemble was trained with all available data and tested with the data available until each day of hospitalization. The first COVID-19 wave is the period between March 2020 and December 2020. The second wave is the period between January 2021 and October 2021

Figure 6 shows that the ensemble trained with all available data and tested with partial data has high accuracy in the second wave (above 80%), even when only the first day of hospitalization was used in the test. For the first wave dataset, the accuracy increased when more days of hospitalization were available, but even with few days of hospitalization used in the test, the model indicates accuracy above 72%.

#### Complete hospitalization history

Table 3 shows that the ensemble trained and tested with the patients’ complete hospitalization history achieved accuracy above 87% for both waves. With this data model, the ensemble correctly predicted most of the discharges. When predicting deaths, the ensemble shows a performance decrease with the second wave dataset, as the specificity is lower than that of the first wave, although it can be still considered high (above 82%).

##### Experiment 1: tests by days of hospitalization with univariate time series models

**Fig. 7:**
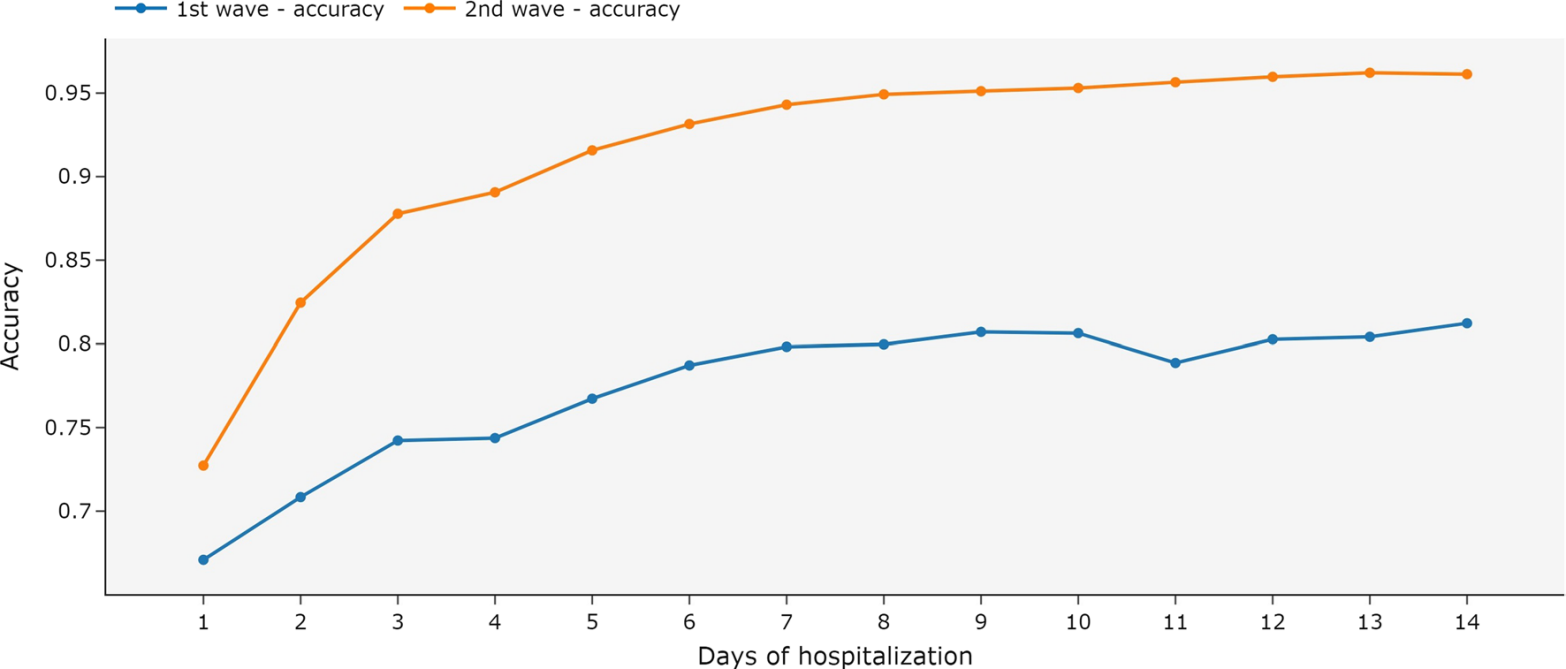
Accuracy for an ensemble of MiniRocket models using complete hospitalization history and univariate time series by day of hospitalization. The ensemble was trained with the complete hospitalization history and tested with the data available until each day of hospitalization. The first COVID-19 wave is the period between March 2020 and December 2020. The second wave is the period between January 2021 and October 2021

In this model, the ensemble was trained with the complete hospitalization history and tested with partial data. The accuracy of the ensemble increased when more data was available in the test (Fig. 7). In the second wave, the accuracy was already above 72% on the first day of hospitalization.

##### Experiment 2: partial hospitalization history with univariate time series models

**Fig. 8:**
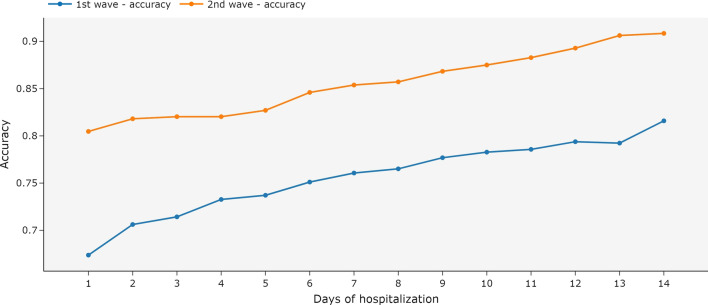
Accuracy for an ensemble of MiniRocket models using complete hospitalization history and univariate time series by day of hospitalization. The ensemble was trained and tested with the data available until each day of hospitalization. The first COVID-19 wave is the period between March 2020 and December 2020. The second wave is the period between January 2021 and October 2021

When trained and tested only with the available vital signs and measurements recorded on the first day of hospitalization, the ensemble achieved accuracies of 67.08% and 72.73% for the first and second waves, respectively. Figure 8 shows that increasing the available hospitalization history in both training and testing steps increases the accuracy of the ensemble. With the second wave dataset, the accuracy was already above 80% on the first day of hospitalization.

### Multivariate time series models

The multivariate time series models use all the eight vital signs/measurements to predict patients’ outcome. We tested these models with different datasets and data models. All metrics, except for those of the experiment one, are average metrics collected during the stratified cross-fold validation.

#### Independent days of hospitalization

Training and testing a multivariate time series model considering independent days of hospitalization resulted in accuracies above 80% (Table 3). In both datasets, the model correctly identified a similar number of deaths. Sensitivity and precision were above 84%.

##### Experiment 1: tests by days of hospitalization with multivariate time series models

**Fig. 9:**
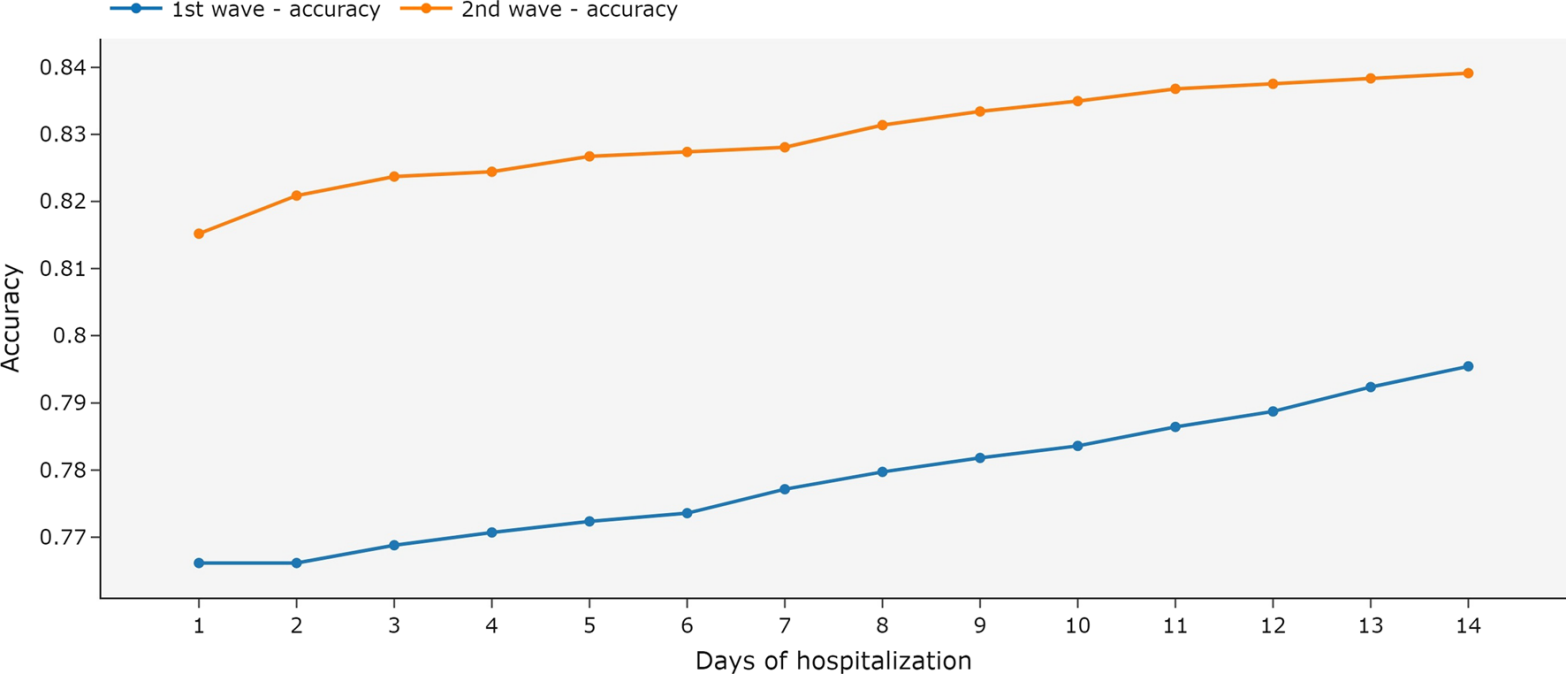
Metrics for MiniRocket models using independent days of hospitalization and multivariate time series. The model was trained with all available data and tested with the data available until each day of hospitalization. The first COVID-19 wave is the period between March 2020 and December 2020. The second wave is the period between January 2021 and October 2021

The multivariate time series achieved higher accuracy as more days of hospitalization were available in the test. With the second wave dataset, even using only the first day of hospitalization, the accuracy of the model was higher (above 81%) when compared with the first wave dataset (Fig. 9), which achieved 76% of accuracy when using only the first day of hospitalization.

#### Complete hospitalization history

A multivariate time series model trained and tested with the complete hospitalization history achieved very high accuracy. As shown in Table 3, the model was capable of correctly predicting both deaths and discharge in both COVID-19 waves, achieving values above 87% for all metrics.

##### Experiment 1: tests by days of hospitalization with multivariate time series models

**Fig. 10:**
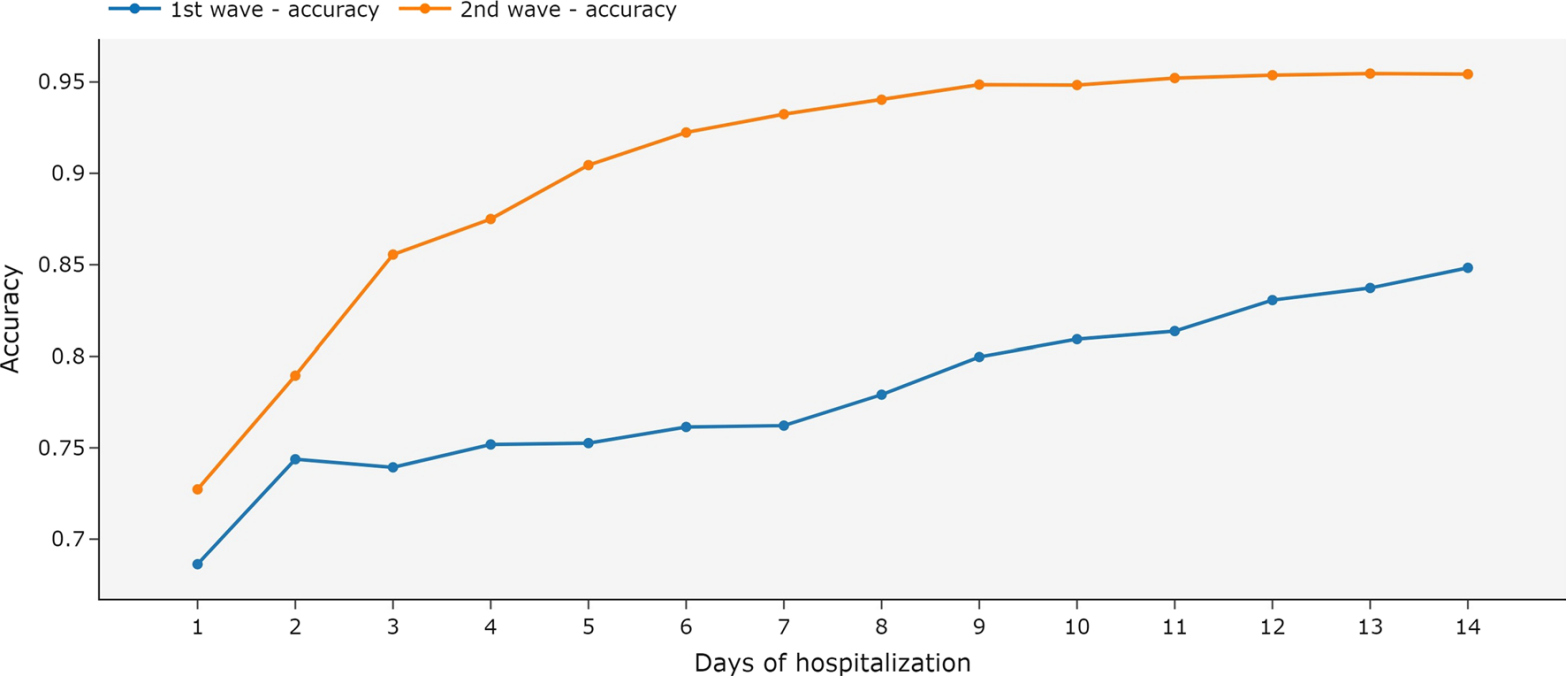
Accuracy for MiniRocket models using complete hospitalization history and multivariate time series by day of hospitalization. The models were trained with the complete hospitalization history and tested with the data available until each day of hospitalization. The first COVID-19 wave is the period between March 2020 and December 2020. The second wave is the period between January 2021 and October 2021

When trained with the complete hospitalization history and tested with partial data, in the second wave, the multivariate time series model achieved accuracies above 70% when using the first day of hospitalization. The accuracy in the second wave became higher as more data was available in the test (Fig. 10), reaching values around 90% in the 5th day and around 95% in the 14th day. Although this same behavior was observed for the first wave dataset, the increase was considerably smaller, reaching values around 85%.

##### Experiment 2: partial hospitalization history with multivariate time series models

**Fig. 11:**
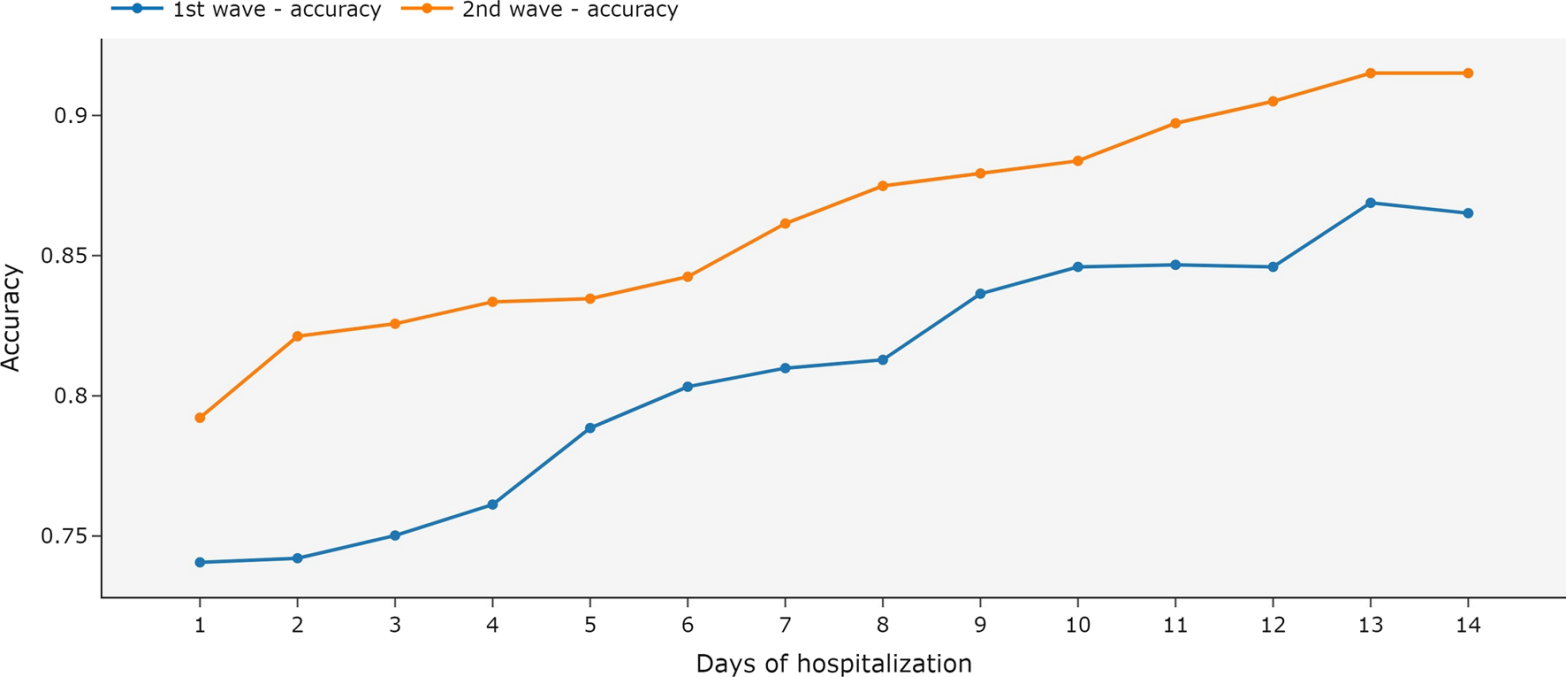
Accuracy for MiniRocket models using complete hospitalization history and multivariate time series by day of hospitalization. The models were trained and tested with the data available until each day of hospitalization. The first COVID-19 wave is the period between March 2020 and December 2020. The second wave is the period between January 2021 and October 2021

For the training and testing with partial data, the multivariate time series model achieved similar accuracies for both waves. Figure 11 shows that as more data were available in the tests, the accuracies increased. Although the behavior is similar to the previous case and the accuracy is similar when all the period is used, the training with partial data accuracy produces smaller accuracies, as can be observed in the middle portion of the curves in the second wave. As for the first wave, accuracy was higher than the previous experiment in the first few days, reaching values around 81% on the seventh day of hospitalization.

### Comparisons between models

**Fig. 12:**
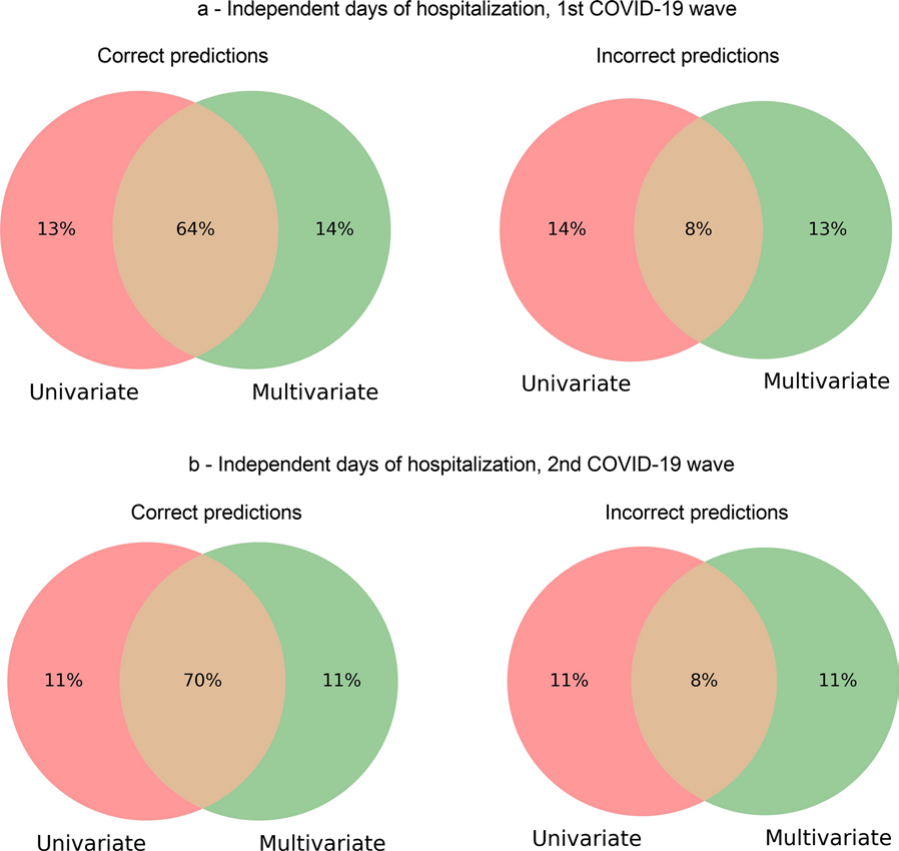
Intersection between predictions made by an ensemble of univariate time series models and by multivariate time series models with independent days of hospitalization. Intersection of correct outcome predictions (left) and incorrect predictions (right) with data regarding the first COVID-19 wave from March 2020 to December 2020 (**a**) and the second wave from January 2021 to October 2021 (**b**)

**Fig. 13:**
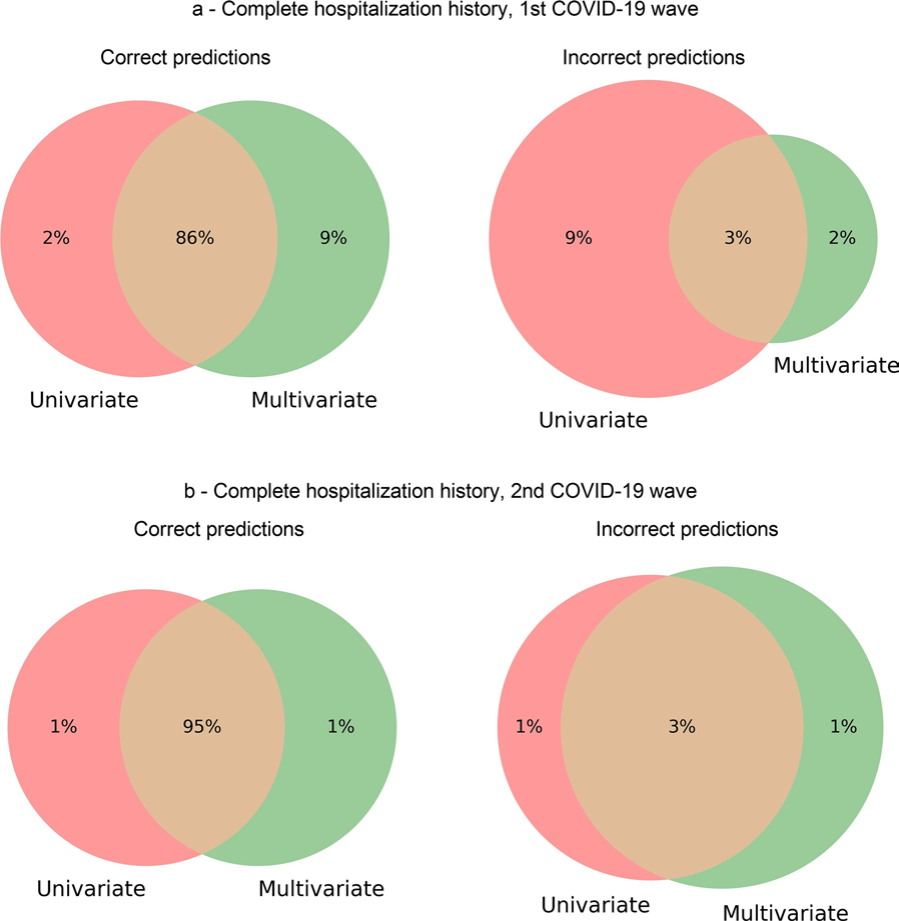
Intersection between predictions made by an ensemble of univariate time series models and by multivariate time series models with complete hospitalization history. Intersection of correct outcome predictions (left) and incorrect predictions (right) with data regarding the first COVID-19 wave from March 2020 to December 2020 (**a**) and the second wave from January 2021 to October 2021 (**b**)

We compared the outcomes predicted by the ensemble and the multivariate models by determining the intersection between predictions. For independent days of hospitalization, in the first wave (Fig. 12a), both approaches predicted the same outcome for 72.89% of patients. In the second wave (Fig. 12b), the predictions coincided for 78.40% of patients. When trained and tested with complete hospitalization history, the model predicted the same outcome for 89.25% and 97.54% of patients in the first (Fig. 13a) and second (Fig. 13b) waves, respectively.

Table 3 shows all results obtained for models trained and tested with independent days of hospitalization and with complete hospitalization history, per COVID-19 wave. These metrics are averages collected during the stratified cross-fold validation with threefolds. In general, models with complete hospitalization history obtained the best results. Depending on the dataset, univariate or multivariate models can achieve higher metrics. The best metrics were obtained when the complete hospitalization history was used to train univariate models.

**Table 3:** Results of all tests conducted with data and machine learning models, with stratified cross-validation

Dataset	Model	COVID-19 wave	Accuracy (%)	Sensitivity (%)	Precision (%)	f1 score (%)	Specificity (%)	Negative predictive value (%)
**Independent days of hospitalization**	**Univariate**	**First**	81.04	*85.16*	86.28	*85.71*	72.75	70.89
		**Second**	83.76	93.15	86.69	89.80	*52.73*	*66.97*
	**Multivariate**	**First**	*80.66*	87.74	*83.98*	85.81	66.52	73.06
		**Second**	83.51	90.56	86.99	88.71	65.80	73.64
**Complete hospitalization history**	**Univariate**	**First**	87.55	86.50	94.94	90.52	**90.60**	76.70
		**Second**	**95.98**	**99.31**	**95.85**	**97.55**	82.29	**96.64**
	**Multivariate**	**First**	94.49	97.75	94.23	95.94	87.96	95.23
		**Second**	95.62	98.65	95.54	97.06	87.43	96.14

The best metrics are bolded and the worst metrics are in italic

## Discussion

The results show that the use of time series to represent EHRs and to predict COVID-19 patients’ outcomes produced robust machine learning models that can reach up to 81% accuracy when only the first hospitalization day is used in the test and all dataset is used in the training. This indicates that by these models one can predict the outcome of the patient with a high accuracy already in the first day of hospitalization. However, it is important to note that the models were trained and tested with an unbalanced dataset, since there are more discharges than deaths. This unbalance is a key factor to the models achieving higher values for sensitivity (discharge prediction) than those for specificity (death prediction).

The data model—independent days or complete hospitalization history—can have a significant impact on the model performance. The best results for all metrics were achieved when using univariate or multivariate models with complete hospitalization history. These results suggest that the MiniRocket method was able to identify patterns in the evolution of the vital signs/measurements during hospitalization that enabled the models to correctly predict more adequately patients’ outcomes.

Although multivariate models are better at correctly predicting both discharges and deaths across datasets, the ensembles of univariate models are designed as “white-boxes” and can offer doctors a more detailed outcome prediction. For example, a patient can have seven vital signs/measurements indicating a discharge, but that one vital sign that indicates death can help doctors make clinical decisions. Thus, doctors can analyze which type of intervention they recommend according to the critical variables identified by the model.

Clinicians started working in the COVID-19 pandemic without any elements to help them, and without evidence to support their decisions [17]. Despite living in a connected world, bedside observations and experience were initially the only sources of guidance.

The natural history of COVID-19 involves a viremic period that lasts approximately a week followed in severe cases by an inflammatory period in which the clinical condition of the patients worsens and death may ensue [18]. Several studies identified risk factors associated with severity and mortality include increased age, multiple preexisting comorbidities, such as cardiovascular disease, hypertension, diabetes mellitus, hypoxia, extension of pulmonary involvement, laboratory tests abnormalities, and biomarkers of end-organ dysfunction [2, 19].

In this study, we evaluated the performance of models to predict outcomes of hospitalized patients using the most simple and accessible patient evaluations which included physical examination; the use of an oximeter; and, when the patient was under mechanical ventilation, breathing rate and ventilator parameters. These models did not require the use of laboratory tests, cardiac monitoring, or imaging. Our results were surprisingly good. There have been other attempts to predict patient outcomes. Use of cardiac monitoring has been attempted to predict severity and mortality [20, 21]. Viral load has been suggested to predict mortality [22]. Severity scores used for prognosis in intensive care, such as qSOFA, NEWS, or SIRS, have shown to perform poorly in COVID-19 [23]. Furthermore, most of these evaluations require data that may not be easily available in all settings. In different areas of the world, conditions of healthcare vary widely [24], which includes differences in access to hospital beds, oxygen, intensive care, advanced respiratory support, laboratory testing, and imaging. The ability to predict the patients’ outcomes may allow proper allocation of high risk patients to more complex care, and our study has shown that it can be done using simple measurements.

All the models presented in our study have great potential to be applied in daily medical practice. For example, the models can be inserted in an application available in mobile devices, in which the patient data are pulled from EHRs database and the model informs the probability of discharge or death in real time. At the same time, the data of each patient with their respective outcome can be used to update the model and improve the metrics.

When comparing the first and the second COVID-19 waves, we found better results in the second wave. Despite having older patients and a higher proportion of patients admitted to the ICU, mortality was lower during the second wave and our models presented a better performance when compared to the first wave. In Brazil, the second wave was due to the SARS-CoV-2 variant Gamma [25]. Although this variant has been shown to be more transmissible [26], other characteristics such as increased virulence have not been demonstrated [27]. However, differences in characteristics of the viruses may explain the difference in performance of the models. Another potential explanation is the enhanced knowledge in COVID-19 management that occurred over the pandemic. Changes in the use of anticoagulants and steroids [28] are examples of this and may have improved the outcome of COVID-19 patients in the second wave, as shown by the lower death rate in our study. Furthermore, knowledge on mechanical ventilation strategies has also improved [29]. These changes in management and patient treatment increased the probability of a positive outcome. Further studies evaluating these factors should clarify differences in outcome and model performance.

Despite the unbalanced datasets, time series models were capable of correctly predicting most of the deaths. The stratified cross-validation strategy did not indicate overfitting in any model. Even so, there are differences in the capabilities of the models for correctly identifying deaths. The ensembles of univariate models had more difficulties predicting deaths, as indicates the 52.73% specificity observed with the second wave dataset and independent days of hospitalization (“Comparisons between models” section).

The biggest difference between univariate and multivariate models can be observed in execution times. Both models need preprocessed data that can be converted to time series. The preprocessing takes up to dozens of minutes. Univariate models with complete hospitalization history take just a few minutes to complete training and testing. Compared to this model, multivariate models with complete hospitalization history take more than 45 times longer to complete the training and testing. In general, training and testing multivariate models takes more time than univariate models (Table 4).

**Table 4:** Table of time needed to complete training and testing time series models according to model type and data model used

Time series model	Data model	Training and testing time
Univariate	Complete hospitalization history	x
Univariate	Independent days of hospitalization	5,62x
Multivariate	Independent days of hospitalization	6,07x
Multivariate	Complete hospitalization history	45,67x

These times refer to the first COVID-19 wave dataset, the biggest available

For both univariate and multivariate models, it is necessary to input a considerable amount of data. In a real world scenario, these models should have access to the EHRs database to collect and preprocess vital signs/measurements data rather than requiring the end user to manually input data. Thus even if univariate models can present a lower performance in some cases, they can be a suitable solution for real time applications.

## Conclusions

Using time series to represent and predict COVID-19 patients’ outcome produces machine learning models with high accuracy and sensitivity for predicting discharges. Even with an unbalanced dataset, the models have good specificity and negative predictive value.

Multivariate time series models offer higher accuracy, sensitivity, and specificity, but take considerable additional time to train and test. An ensemble of univariate time series models takes considerably lower times to be trained and tested and offers a detailed outcome prediction for each of the available vital signs. However, the ensemble will have more difficulties correctly predicting deaths.

These high accuracy models based on vital signs/measurements can be used as a support for clinical decisions. The fact that these models used data that is very easily obtainable, without requiring laboratory tests or imaging, makes them even more promising for use in a variety of healthcare settings, especially if a simple and accessible application can be developed for mobile phones or personal computers. Finally, it is possible that, with available data, these models can potentially predict outcomes for other diseases, requiring further studies.

## Data Availability

The datasets analyzed during the current study are not publicly available, since they were extracted from Hospital das Clí­nicas patients’ electronic health records. Data on patients are protected by medical confidentiality. Although, we removed all identifiers from the dataset, we believe that identities may still not be completely protected because individual patient data such as age, sex, dates of hospital admission and discharge, and descriptions of medical conditions may make identification of patients still possible. The ethics approval includes the assurance that patient data will be analyzed in aggregate and that individual patient data will not be released. Data requests can be addressed to the authors, who will evaluate the possibility of fulfilling the request considering the patients’ privacy.

## Abbreviations

CI: Central Institute
COVID-19: Coronavirus disease 2019
EHR: Electronic health records
HC: Hospital das Clínicas
MiniRocket: MINImally RandOm Convolutional KErnel Transform
